# Familial cancer aggregation and the risk of lung cancer

**DOI:** 10.1590/S1516-31802002000200003

**Published:** 2002-03-02

**Authors:** Victor Wünsch-Filho, Paolo Boffetta, Didier Colin, José Eduardo Moncau

**Keywords:** Lung cancer, Familial aggregation, Smoking, Case-control study, Interaction, Câncer pulmão, Câncer na família, Tabagismo, Estudos caso-controle, Interação

## Abstract

**CONTEXT:**

Around 90% of lung cancer worldwide is attributable to cigarette smoking, although less than 20% of cigarette smokers develop lung cancer. Other factors such as diet, chronic lung diseases, occupation and possibly environmental agents also contribute to this cancer. Genetic factors seem to play a role in lung cancer, but the precise characteristics influencing lung cancer susceptibility are not known, since genetic factors are easily obscured by the strong environmental determinants of lung cancer, particularly smoking.

**OBJECTIVE:**

To estimate the effect that cancer occurrence among first-degree relatives has on the risk of lung cancer.

**DESIGN:**

Hospital-based case-control study.

**SETTING:**

The metropolitan region of São Paulo, Brazil.

**PARTICIPANTS:**

334 incident lung cancer cases and 578 controls matched by hospitals.

**MAIN MEASUREMENTS:**

By means of a structured questionnaire, cases and controls were interviewed about cancer occurrence in first-degree relatives, tobacco smoking, exposure to passive smoking, occupation, migration and socioeconomic status. Non-conditional logistic regression was used to calculate the risk of familial cancer aggregation, the effect of cancer in first-degree relatives and smoking in conjunction, and for controlling confounders.

**RESULTS:**

The adjusted odds ratio (OR) revealed a slight, but not statistically significant, excess risk of lung cancer for subjects with a history of lung cancer in relatives (OR 1.21; 95% confidence interval [CI] 0.50 – 2.92). The same was found among those with a history of other tobacco-related cancers in relatives (OR 1.36; 95% CI 0.87 – 2.14). A step gradient effect was observed regarding lung cancer risk, in accordance with increases in the number of pack-years of cigarette consumption. An interaction between familial cancer aggregation and tobacco smoking was detected.

**CONCLUSIONS:**

A mildlyelevated risk of lung cancer among persons with a positive history of lung and other tobacco-related cancers was observed. The finding of an interaction between the variables of familial cancer aggregation and smoking suggests that familial cancer aggregation could be considered as a marker of susceptibility, increasing the risk of lung cancer among smokers. These results improve our knowledge of lung carcinogenesis and can guide future cancer genetic studies.

## RESUMO

**INTRODUÇÃO:**

Cerca de 90% dos casos de câncer de pulmão no mundo são atribuíveis ao tabagismo, porém menos de 20% dos fumantes desenvolvem câncer de pulmão. Fatores como dieta, doenças pulmonares crônicas, ocupação e, possivelmente, exposições ambientais também têm papel na etiologia desse câncer. Os fatores genéticos parecem influir na ocorrência da doença, mas as características que influenciam a suscetibilidade à neoplasia pulmonar não são precisamente conhecidas, obscurecidas pela forte influência dos fatores ambientais na determinação da doença, particularmente o tabagismo.

**OBJETIVOS:**

Estimar o efeito da ocorrência de câncer em parentes de primeiro grau no risco de câncer de pulmão.

**TIPO DE ESTUDO:**

Estudo caso-controle de base hospitalar.

**LOCAL:**

Região Metropolitana de São Paulo.

**PARTICIPANTES:**

334 casos de neoplasia pulmonar e 578 controles emparelhados por hospital.

**VARIÁVEIS ESTUDADAS:**

Casos e controles foram entrevistados com respeito ao passado de neoplasias em parentes de primeiro grau, tabagismo, tabagismo passivo, ocupação, migração e status socioeconômico. Utilizouse a regressão logística não-condicional para calcular o risco de câncer em familiares, o efeito conjunto de câncer em familiares e uso de tabaco, e para controlar potenciais variáveis de confusão.

**RESULTADOS:**

O *odds ratio* (OR) ajustado revelou um discreto excesso de risco para câncer de pulmão, não estatisticamente significante, entre os indivíduos com história de câncer de pulmão na família (OR 1,21; intervalo de confiança de 95% [IC 95%] 0,50 – 2,92) ou entre aqueles com história de outros cânceres relacionados ao tabaco na família (OR 1,36; IC 95% 0,87 –2,14). Foi observado um efeito dose-resposta positivo para o risco de câncer de pulmão de acordo com o aumento do consumo de cigarros. Detectou-se uma interação entre as variáveis câncer na família e tabagismo.

**CONCLUSÕES:**

Observou-se um discreto aumento do risco de câncer de pulmão entre indivíduos com história positiva na família de câncer de pulmão e outros cânceres relacionados ao tabaco. Esses resultados sugerem que a presença de aglomerados de câncer na família pode ser considerada como um marcador de suscetibilidade e aumenta o risco de câncer de pulmão entre os fumantes. A interação detectada entre as variáveis agregadas de câncer na família e tabagismo no risco de câncer de pulmão é uma contribuição no conhecimento dos mecanismos da carcinogênese e poderá orientar futuras pesquisas no campo da genética do câncer.

## INTRODUCTION

The majority of lung cancer cases, about 90%, can be attributed to cigarette smoking,^1^ but no more than 20% of smokers develop lung cancer.^2^ The prevalence of tobacco usage is on the increase in developing countries.^3^ Other factors, such as diet, chronic lung diseases, occupational and possibly environmental agents have been shown to contribute to the development of this cancer.^4^ Genetic factors seem to play a role in the causality of lung cancer, but the precise characteristics influencing lung cancer susceptibility are not known. Such genetic factors are easily obscured by the strong environmental determinants of lung cancer, particularly smoking.^4^

Various studies have shown elevations in the risks for different cancers among those who reported cancer in relatives.^5-10^ Evidence of some degree of familial aggregation of lung cancer is brought out in most familial studies. Since the pioneering study of Tokuhata and Lilienfeld^11^ on familial aggregation of lung cancer, some studies have consistently demonstrated an increased prevalence of cancer among relatives of lung cancer patients, with risks varying from 1.3 to 12.2.^12-22^ However, other studies concluded that there was little evidence of familial cancer clusters related to lung cancer risk.^23,24^ Some authors have suggested that a genetic predisposition to lung cancer may contribute to familial aggregation of this cancer.^2,20,22,25^ In addition, studies of familial cancer aggregation may provide important insights into the understanding of the interplay between environmental and genetic risk factors in cancer development.

Lung cancer is the most common neoplastic disease among Brazilian males and the main cause of death from cancer. Since the 1970s the incidence and mortality rate of lung cancer have been rising, and during the 1990s have been increasing among females.^26^ This pattern has been associated with an increase in the prevalence of tobacco smoking.^27,28^ No studies have been published on familial cancer aggregation and lung cancer in Brazil. However the results of a case-control study conducted in Rio de Janeiro showed that polymorphism of the Cyp1A1 gene, encoding for an enzyme involved in the metabolism of tobacco carcinogens, was associated with lung cancer risk.^29^

The objective of this study was to examine the effect that reported histories of cancer among first-degree relatives had on lung cancer among the participants in a case-control study carried out in the metropolitan region of São Paulo (MRSP), an area with high incidence and mortality rates of lung cancer.

## METHODS

A hospital-based case-control study was structured so as to detect incident cases of primary lung cancer among males and females in 14 hospitals where the majority of lung cancer cases in the MRSP are admitted. Information was gathered on cancer in first-degree relatives (parents, siblings and offspring), tobacco smoking, passive smoking in childhood and adult life, socioeconomic status, occupational exposures and medical history. Some additional details on the structure of the study and analysis of occupational risk factors have already been described in a previous publication.^30^

A total of 912 persons were included in the analysis, of whom 334 were cases and 578 controls. The cases chosen for the study were newly diagnosed as having lung cancer, according to the International Classification of Disease, ninth revision (ICD-9),^31^ rubric 162, between January 1989 and June 1991. The diagnosis was assessed from hospital records and only cases confirmed by histology or cytology were accepted. Cases confirmed by cytology were assumed to be of undifferentiated histological type. Eligible cases with a definite diagnosis other than lung cancer were reclassified as controls whenever the diagnosis was among those retained as admissible for controls. Only patients who had been resident for at least six months in the MRSP were included, and all patients were interviewed in person. Controls were enrolled from the same hospitals and during the same period as the cases, and also had to have been resident in the MRSP for at least six months. To determine the controls’ eligibility, their diagnoses abstracted from medical records were coded according to the ICD-9. Patients with chronic obstructive respiratory diseases, circulatory diseases and smoking-related cancers (bladder, larynx, esophagus, oral cavity, pharynx, pancreas, kidney and renal pelvis) were excluded.

A standard questionnaire specifically drawn up for the gathering of information on the study variables (family cancer history, tobacco smoking, passive smoking, occupation, migration and socioeconomic status) was applied to both cases and controls. Family cancer history was recorded regarding any first-degree relative (mother, father, sibling, son or daughter) having a cancer at any anatomical site. By means of a structured interview, the health status of each first-degree relative, including the disease diagnosis of sick relatives and the cause of death of deceased relatives were recorded. Pathological confirmation of cancer, and smoking habits of relatives were not obtained.

Odds ratio (OR) and 95% confidence intervals (CI) were calculated as approximations of relative risk.^32^ The risk of lung cancer was estimated by comparing subjects reporting a positive history of cancer in first-degree relatives with those who did not. Cases and controls were stratified according to the number of cancer cases in first-degree relatives and the proportion of reported cases over the total number of relatives. The risk of lung cancer was also estimated for different groups of neoplasm among relatives: lung cancer, tobacco-related cancers other than lung cancer, and other cancers not related to tobacco. The analysis of tobacco smoking was based on categories of cumulative consumption expressed in pack-years. The OR and the 95% CI estimates were obtained by non-conditional logistic regression using the maximum likelihood estimate, adjusting for sex, age, smoking and socioeconomic status. Socioeconomic status was classified into four levels based on a combination of income and level of education.

## RESULTS

Selected characteristics of cases and controls are shown in Table 1. Of the 334 lung cancer cases 76.6% were men and among the 578 hospital controls 64.2% were men. The age range of cases was 36-90 years, and for controls 33-90 years. In males, squamous cell carcinoma was the most frequent histological type and in females, adenocarcinoma was predominant. Only 28 lung cancer cases (8.4%) did not have diagnoses established by histology. The distribution of lung cancer cases according to age, gender and histological type of tumor was similar to what has been reported in previous studies in Brazil^33^ and worldwide.^4^ The main diagnoses for controls were: infectious and parasitic diseases (23.5%), non-tobacco related neoplasms (17.5%), diseases of the digestive system (15.1%), endocrine, nutritional, metabolic diseases and immune disorders (7.4%). The remaining controls had diverse diseases such as bone fracture, rheumatoid arthritis, skin diseases, and in women, childbirth complications. Males had a higher proportion of infectious diseases and a lower proportion of neoplasms than did females.

**Table 1:** Selected characteristics of cases and controls by sex

CHARACTERISTIC	MALES		FEMALES	
	Cases (n = 207) n (%)	Controls (n = 371) n (%)	Cases (n = 78) n (%)	Controls (n = 256) n (%)
**AGE^1^**				
< 50 years	25 (9.8)	75 (20.2)	11 (14.1)	31 (15.0)
50-59 years	77 (30.1)	119 (32.1)	27 (34.6)	54 (26.1)
60-69 years	95 (37.1)	112 (30.2)	29 (37.2)	82 (39.6)
≥ 70 years	59 (23.0)	65 (17.5)	11 (14.1)	40 (19.3)
**HISTOLOGICAL TUMOR TYPE**				
Squamous cell carcinoma	120 (46.9)	–	22 (28.2)	–
Adenocarcinoma	59 (23.0)	–	29 (37.2)	–
Small cell carcinoma	36 (14.1)	–	12 (15.4)	–
Large cell carcinoma	11 (4.3)	–	2 (2.5)	–
Other, mixed and undifferentiated^2^	30 (11.7)	–	13 (16.7)	–
**DIAGNOSES OF CONTROLS (ICD-9 codes^3^)**				
Infectious and parasitic diseases (001 - 139)	–	111 (29.9)	–	25 (12.1)
Non-tobacco related neoplasms (140 - 239)	–	43 (11.6)	–	58 (28.0)
Endocrine, nutritional and metabolic diseases and immune disorders (240 – 279)	–	20 (5.4)	–	23 (11.1)
Diseases of the digestive system (520 - 579)	–	50 (13.5)	–	37 (17.9)
Other diagnoses	–	147 (39.6)	–	64 (30.9)

1 Age range: 36-90 for cases; 33-90 for controls.

2 Including all tumors confirmed by cytology.

3 ICD-9 = International Classification of Diseases, Ninth Edition.^31^

From a combination of *per capita* income and level of education, four socioeconomic tiers were established. Males in the highest socioeconomic level showed a slightly higher but non-significant risk of developing lung cancer than did those in the lowest tier (OR 1.34; 95% CI 0.79 – 2.29), with this difference being more pronounced among females (OR 3.75; 95% CI 1.36 – 10.29).

The prevalence of any cancer in first-degree relatives among cases (27.2%) was higher than among controls (21.3%), but no statistically significant association was found (OR 1.16; 95% CI 0.82 – 1.65). Nor was any correlation found between the risk of lung cancer and number of cancers among first-degree relatives (Table 2). A slight excess risk was found within the smokers group (OR 1.24; 95% CI 0.81 – 1.90), and for nonsmokers (OR 1.53; 95% CI 0.61 – 3.83), among those for whom 10% or more of their first-degree relatives had a positive history of cancer (Table 3). The risk of lung cancer according to type of cancer, as reported by category of relatives, did not reveal any significant odds ratio (Table 4). As Table 5 demonstrates, there were elevated risks among subjects whose relatives had lung cancer (OR 1.73; 95% CI 0.75 – 3.96) and other tobacco-related cancers (OR 1.67; 95% CI 1.11 – 2.53). However, controlling for smoking dramatically affected the risk for those reporting lung cancer among relatives (OR 1.21; 95% CI 0.50 – 2.92), or other tobacco-related cancers (OR 1.36; 95% CI 0.87 – 2.14).

**Table 2:** Odds ratio for lung cancer according to the number of cancer cases among first-degree relatives

Cancer in relatives^1^	Cases (n = 334)	Controls (n = 578)	OR^2^ (95% CI)
None^3^	230	429	1.0
Any	91	123	1.16 (0.82 – 1.65)
1 case	73	95	1.26 (0.86 – 1.84)
2 cases	15	22	0.96 (0.47 – 1.99)
≥ 3 cases	3	6	0.60 (0.13 – 2.83)

1 Any cancer in parents and siblings

2 Odds ratio adjusted for gender, age, socioeconomic status (four levels) and smoking (pack-years)

3 Reference category.

**Table 3:** Odds ratio for lung cancer according to the percentage of first-degree relatives with cancer

Percentage of cancer in relatives^1^	Cases (n = 334)	Controls (n = 578)	OR^2^ (95% CI)
**TOTAL^3^**			
< 10%	23	31	1.15 (0.61 – 2.15)
≥ 10%	65	87	1.12 (0.75 – 1.67)
**SMOKERS^3^**			
< 10%	22	19	1.37 (0.71 – 2.67)
≥ 10%	57	55	1.24 (0.81 – 1.90)
**NONSMOKERS^3^**			
< 10%	1	12	0.44 (0.05 – 3.71)
≥ 10%	8	32	1.53 (0.61 – 3.83)

1 Any cancer in parents and siblings

2 Odds ratio adjusted for gender, age, socioeconomic status (four levels); and smoking (pack-years) for the total group

3 Reference category: no cancer reported among relatives.

**Table 4:** Odds ratio for lung cancer according to the number of cancer cases by category of first-degree relatives

Cancer in first-degree relatives^1^	Cases (n = 334)	Controls (n = 578)	OR^2^ (95% CI)
None^3^	230	429	1.00
**Mother**			
Any	27	43	1.10 (0.64 – 1.91)
Not tobacco-related	10	19	0.88 (0.38 – 2.04)
Tobacco-related^4^	14	19	1.37 (0.64 – 2.94)
Lung	3	5	1.01 (0.21 – 4.97)
**Father**			
Any	28	43	1.09 (0.63 – 1.89)
Not tobacco-related	8	17	0.87 (0.34 – 2.19)
Tobacco-related^4^	16	19	1.48 (0.70 – 3.13)
Lung	4	7	0.68 (0.18 – 2.53)
**Siblings**			
Any	52	61	1.20 (0.76 – 1.87)
Not tobacco-related	14	21	1.10 (0.52 – 2.35)
Tobacco-related^4^	31	33	1.29 (0.73 – 2.30)
Lung	7	7	1.05 (0.34 – 3.21)

1 No cancer was reported in the offspring

2 Adjusted for gender, age, socioeconomic status (four levels), smoking (pack-years), and number of siblings (group of siblings)

3 Reference category

4 Bladder, larynx, esophagus, oral cavity, pharynx, pancreas, kidney and renal pelvis.

**Table 5:** Odds ratio for lung cancer according to the type of cancer in first-degree relatives

Type of cancer in relatives^1^	Cases (n = 334)	Controls (n = 578)	OR^2^ (95% CI)
**OR adjusted for gender, age and socioeconomic status^2^**			
No cancer^3^	239	452	1.0
Not tobacco-related cancers	30	54	1.02 (0.63 – 1.66)
Tobacco-related^4^	53	60	1.67 (1.11 – 2.53)
Lung	12	12	1.73 (0.75 – 3.96)
**OR adjusted for gender, age, socioeconomic status and smoking^5^**			
No cancer^3^	230	429	1.0
Not tobacco-related cancers	29	53	0.93 (0.55 – 1.57)
Tobacco-related^3^	50	58	1.36 (0.87 – 2.14)
Lung	12	12	1.21 (0.50 – 2.92)

1 Parents and siblings

2 Considering four levels, from level 1 (lowest level) to level 4 (highest level), based on a combination of income per capita and level of education

3 Reference category

4 Bladder, larynx, esophagus, oral cavity, pharynx, pancreas, kidney and renal pelvis

5 Pack-years.

Tobacco smoking was an important risk factor for lung cancer and a step gradient effect was observed in relation to increasing numbers of pack-years of cigarettes smoked, which was stratified into five groups (Table 6). The increase in the risk can be seen from the reports of cancer in relatives for each cumulative consumption pack-years stratum, except for the 1-20 pack-year stratum.

**Table 6:** Joint effect on lung cancer risk of smoking (5 strata) and cancer among first-degree relatives (2 strata)

Smoking (pack-years)	Any cancer in relatives^1^	Cases (n = 334)	Controls (n = 578)	OR^2^ (95% CI)
Nonsmoker	No	28	162	1.00
	Yes	9	47	1.08 (0.47 – 2.47)
1 – 20	No	39	88	3.36 (1.87 – 6.03)
	Yes	6	24	1.86 (0.68 – 5.10)
21 – 40	No	50	85	5.18 (2.80 – 9.58)
	Yes	17	19	7.17 (3.19 – 16.13)
41 – 60	No	55	49	8.93 (4.78 – 16.65)
	Yes	29	18	11.73 (5.44 – 25.25)
≥ 61	No	58	45	10.03 (5.32 – 18.91)
	Yes	30	15	14.90 (6.68 – 33.25)

1 Parents and siblings

2 Adjusted for gender, age and socioeconomic status (four levels).

Table 7 shows an analysis of smoking according to two strata of cumulative consumption (0-20 and 21 or more pack-years), combined with the dichotomous variable of cancer among relatives. No effect of familial cancer aggregation was detected when there was a consumption of less than 20 pack-years, which suggests an interaction between smoking and familial cancer aggregation. Further stratification by gender or age made no difference to the detection of this interaction (Table 8).

**Table 7:** Joint effect on lung cancer risk of smoking (2 strata) and cancer among first-degree relatives (2 strata)

Smoking (pack-years)	Cancer in relatives^1^	Cases (n = 334)	Controls (n = 578)	OR^2^ (95% CI)
0 – 20	No^3^	67	250	1.00
	Yes	15	71	0.79 (0.42 – 1.47)
≥ 21	No	163	179	3.53 (2.49 – 5.01)
	Yes	76	52	5.29 (3.37 – 8.30)

1 Any cancer in parents and siblings

2 Odds ratio adjusted for age and socioeconomic status (four levels)

3 Reference category.

**Table 8:** Joint effect on lung cancer risk of smoking (2 strata) and cancer among first-degree relatives (2 strata), by gender and age

	Smoking (pack-years)	Cancer in relatives^1^	Cases (n = 334)	Controls (n = 578)	OR^2^ (95% CI)
***GENDER***					
Males	0 – 20	No^3^	29	120	1.00
		Yes	4	29	0.50 (0.16 – 1.56)
	≥ 21	No	148	163	3.84 (2.39 – 6.16)
		Yes	66	45	5.50 (3.13 – 9.68)
Females	0 – 20	No^3^	38	130	1.00
		Yes	11	42	0.93 (0.43 – 2.04)
	≥ 21	No	15	16	3.25 (1.42 – 7.42)
		Yes	10	7	4.89 (1.68 – 14.25)
***AGE***					
< 60 years	0 – 20	No^3^	30	122	1.00
		Yes	7	27	1.00 (0.39 – 2.56)
	≥ 21	No	72	100	3.66 (2.07 – 6.46)
		Yes	27	24	4.96 (2.45 – 10.07)
≥ 60 years	0 – 20	No^3^	37	128	1.00
		Yes	8	44	0.62 (0.27 – 1.45)
	≥ 21	No	91	79	3.86 (2.25 – 6.63)
		Yes	49	28	5.74 (2.97 – 11.09)

1 Parents and siblings

2 Odds ratio adjusted for age, gender and socioeconomic status (four levels)

3 Reference category.

## DISCUSSION

The objective of this study was to examine how familial cancer aggregation affects the risk of lung cancer. Evidence for the genetic basis of cancer has increased over recent decades and thus an assessment of familial cancer aggregation may play an important role in epidemiological studies. Excessive frequency of a trait or disorder within specific families as compared with the population at large is the focus of familial aggregation studies. Various cancer studies have noted this relationship, but the main problem arising from the results of epidemiological studies lies in the difficulty in balancing the influence of heredity versus shared environmental factors.^34^ Inter-individual variation in response to xenobiotics and their potential carcinogenic effects could be mediated by inherited genetic predisposition, although familial aggregation may also be explained by the fact that people of the same family tend to share the same habits, such as tobacco smoking, alcohol drinking, diet and occupation. The ability to disentangle the role of a genetic/familial component from the effect of tobacco smoking on lung cancer risk can improve our knowledge of lung carcinogenesis and have an impact on cancer prevention.

Several studies of familial aggregation of lung cancer have suggested an underlying genetic susceptibility.^2,11-13,15-22^ Sellers et al.^14^ provided strong evidence of Mendelian inheritance in lung cancer, but the study by Yang et al.^35^ rejected the Mendelian model as an explanation of lung cancer occurrence.

The most recent publications of population-based studies of familial aggregation of lung cancer used the strategy of investigating only nonsmokers.

The study by Schwartz et al.^17^ suggested the hypothesis of genetic susceptibility to lung cancer, since the familial aggregation was found only among the relatives of younger nonsmoking lung cancer cases and among younger relatives. Similar results were found by Kreuzer et al.^19^ and Yang et al.^36^ Wu et al.^16^ studied only female nonsmokers and found no association between family history of cancer and risk of lung cancer among nonsmokers, but did suggest that the risk of lung cancer increased among females with a female relative (mother or sister) with lung cancer. However, the lack of additional information about lifestyle risk factors among the family members restricted the interpretation of results of that study. Brownson et al.^18^ investigated nonsmokers and ex-smokers and, noting a slight increase in risk, they suggested an interaction between genetic susceptibility and smoking due to the increased chances of lung and oral cavity cancer among former smokers. Yang et al.^35^ studied nonsmoking males and females, and suggested that the pattern of lung cancer occurrence in families of non-smoking lung cancer patients differs from that in families of smoking lung cancer patients. This was based on the fact that the mean age for the onset of lung cancer among female relatives was 55 years for smokers and 88 years for nonsmokers.

Such results are suggestive of the presence of a high-risk gene contributing to early-onset lung cancer in a population where the pro-bands are nonsmokers. Nevertheless, these results still do not clarify whether the evidence supporting a familial association suggests that the etiology of lung cancer includes shared genes or shared environments, or both of these. Certainly, environment factors such as passive smoking must have some influence on lung cancer occurrence in the family. However, many other non-environmental mechanisms might provide additional explanations. Such mechanisms may include common genetic polymorphism of carcinogen-metabolizing enzymes, mutations of tumor suppressor genes or variability in DNA repair activity.^37^

The results from this study have revealed a slight, but non-statistically significant, excess lung cancer risk among those who reported tobacco-related cancer cases and lung cancer among first-degree relatives after controlling for the confounding effect of tobacco (Table 5). These results are contrary to those risks found in the classical case-control studies on the influence of familial cancer aggregation factors on lung cancer.^11,12^ However, they are in general compatible with the low excess risk found by Shaw et al.,^13^ Wu et al.,^16^ Brownson et al.^18^ and Gupta et al.^21^

The magnitude of the risk from familial cancer aggregation was higher among nonsmokers than for smokers, but the odds ratios were not statistically significant (Table 3). However, when the influence of smoking on subjects whose relatives had cancer was examined, an interaction was detected (Table 6 and 7).

Kreuzer et al.^19^ detected remarkable differences in lung cancer risk between probands in younger age groups who reported cancer in relatives or not, but not for older age groups. This finding is also supported by the work of Gauderman and Morrison.^25^ In our study there was no difference in the interaction of cigarette smoking with familial cancer aggregation according to age group (< 60 and 60 years and over) or gender (Table 8).

The results of this study may have been affected by bias. There may have been interference in family history investigations caused by misclassification. Geographical separation limits the availability of information on family histories of disease, thus resulting in an underreporting of familial cases.^38^ Moreover, positive histories may be inaccurate because metastatic cancer sites can often be mistaken for primary ones. In this study, cancer diagnoses among relatives were not histologically confirmed. Love et al.^39^ estimated the accuracy of family cancer history reports and concluded that information on primary-site cancer among first-degree relatives was 83% accurate.

The lack of data on relatives’ ages may also have distorted the estimates, considering that subjects with older relatives would be expected to have more cancer in the family than would subjects with younger relatives. In an effort to correct for this impediment, we examined the risk by category of first-degree relative. However, no difference in lung cancer risk was noted from reports of cancer occurrences among mothers, fathers or siblings (Table 4).

Another major problem in this study was the lack of data on smoking among first-degree relatives. Cancer among relatives is likely to be related to smoking exposure within the family and thus, without this information, a familial/genetic effect cannot be clearly separated from the smoking effect. Table 5 shows how the control for smoking exposure affected the OR of the association between cancer among first-degree relatives and lung cancer. This suggests that incorporating information on relatives who smoke might further reduce the observed association.

Use of hospital controls may have caused selection bias, since 11.6% of male controls and 28.0% of female controls were cancer patients. If a positive familial history of cancer was a risk factor for cancers at sites other than the lung, then the choice of cancer controls would have resulted in a bias towards nullity. However, the exclusion of cancer controls did not modify the results substantially.

Lack of statistical power may have been another potential and additional limitation to this study. The predominantly null results presented in Tables 2 to 5 may be due to this fact.

Despite these possible limitations, the findings showing a mild effect of familial aggregation cancers suggest that common susceptibility genes may act to increase the risk of lung cancer. Reinforcing this, the stratified analysis suggested evidence of an interaction between a positive history of neoplasm in the family and tobacco smoking (Tables 6 and 7). Two facts could possibly explain these interaction findings: (a) residual confounding in the category of 20 and more pack-years; or (b) genetic or environmental familial factors acting on some carcinogenic pathways relevant only for heavy smokers, such as detoxification or damage repair above a certain threshold.

Tobacco smoking, the main environmental risk factor for lung cancer, is strictly dependent on cultural and demographic characteristics. Recent studies have suggested that the initiation of smoking and nicotine dependence may be linked to genetic factors.^40^ There is considerable variation in individuals’ susceptibility to lung cancer, and it has been postulated that this is due to genetic polymorphisms of carcinogen metabolizing enzymes.^41^ However, no clear mechanisms have been established to explain this relationship of interactions between genetic polymorphisms and tobacco carcinogen metabolism, and their further effects on lung cancer. For example, glutathione S-transferase (GST) null genotypes seem to be related to a higher incidence of polycyclic aromatic hydrocarbon DNA adducts and a higher risk of lung cancer,^42-45^ yet some studies have found no such associations.^46^ One study showed an increased risk of lung cancer among individuals combining GST null genotype and a smoking consumption of at least 35 pack-years.^47^ Certainly, many tobacco carcinogens are metabolized by enzymes of the P450 cytochrome family and GST family. Polymorphisms of cytochrome P450, Cyp1A1 and Cyp1A2, and the GST isoenzyme GSTM1 could have implications for polycyclic aromatic hydrocarbon metabolism.^48^ This may explain the increases in the probability of lung cancer among smokers of more than 20 pack-years, as found in this study.

## CONCLUSION

This study reports results from a hospital-based case-control study showing a mildly elevated risk of lung cancer among persons with a positive family history of lung and other tobacco-related cancers. The results suggest that familial cancer clusters may have some connections with tobacco smoking. The finding of an interaction between the variables of familial cancer aggregation and cigarette smoking on the risk of lung cancer may guide future genetic studies and improve our knowledge of lung cancer carcinogenesis.
